# Brain Isoform Glycogen Phosphorylase as a Novel Hepatic Progenitor Cell Marker

**DOI:** 10.1371/journal.pone.0122528

**Published:** 2015-03-31

**Authors:** Yu-Wen Huang, Chien-Chang Chiu, Ja-Der Liang, Ling-Ling Chiou, Guan-Tarn Huang, Ming-Jiun Yu, Hsuan-Shu Lee

**Affiliations:** 1 Institute of Biotechnology, National Taiwan University, Taipei, Taiwan; 2 Division of Medical Devices and Cosmetics, Food and Drug Administration, Ministry of Health and Welfare, Taipei, Taiwan; 3 Department of Internal Medicine, National Taiwan University, Taipei, Taiwan; 4 Liver Disease Prevention and Treatment Research Foundation, Taipei, Taiwan; 5 Institute of Biochemistry and Molecular Biology, National Taiwan University College of Medicine, Taipei, Taiwan; 6 Agricultural Biotechnology Research Center, Academia Sinica, Taipei, Taiwan; Academia Sinica, TAIWAN

## Abstract

An appropriate liver-specific progenitor cell marker is a stepping stone in liver regenerative medicine. Here, we report brain isoform glycogen phosphorylase (GPBB) as a novel liver progenitor cell marker. GPBB was identified in a protein complex precipitated by a monoclonal antibody Ligab generated from a rat liver progenitor cell line Lig-8. Immunoblotting results show that GPBB was expressed in two liver progenitor cell lines Lig-8 and WB-F344. The levels of GPBB expression decreased in the WB-F344 cells under sodium butyrate (SB)-induced cell differentiation, consistent with roles of GPBB as a liver progenitor cell marker. Short hairpin RNA (shRNA)-mediated GPBB knockdown followed by glucose deprivation test shows that GPBB aids in liver progenitor cell survival under low glucose conditions. Furthermore, shRNA-mediated GPBB knockdown followed by SB-induced cell differentiation shows that reducing GPBB expression delayed liver progenitor cell differentiation. We conclude that GPBB is a novel liver progenitor cell marker, which facilitates liver progenitor cell survival under low glucose conditions and cell differentiation.

## INTRODUCTION

Pluripotent progenitor cells are critical elements in regenerative medicine. Many progenitor cells were developed for various tissues including the liver: oval cells [1–3], liver epithelial cells [4–9] and small hepatocyte-like cells [10]. Advances in liver progenitor cell research may lead to new cell therapies and facilitate the development of new drugs [11–13]. However, many of the liver progenitor cells were very hard to isolate due to limited liver progenitor cell markers. Thus, a proper liver progenitor cell marker is highly desirable to accelerate the development of liver regenerative medicine.

We have previously derived an adult hepatic progenitor cell line Lig-8 from the non-parenchymal fraction of liver cells prepared from Fischer 344 rats [14, 15]. The Lig-8 cells share many properties of the well-known liver progenitor cells WB-F344 [4–7] including epitheloid morphology, growth, and expression of hepatocyte or cholangiocyte markers: alpha fetal protein (AFP), albumin, alpha 1-antitrypsin, H.4 antigen, cytokeratin 8, cytochrome P 450 and cytokeratin 7 [4, 16, 17]. These cells can differentiate bi-potentially into hepatocyte- or cholangiocyte-lineage cells following induction by sodium butyrate (SB), a histone deacetylase inhibitor known to affect gene expression, inhibit proliferation and induce differentiation [6, 17, 18].

To identify potential liver progenitor cell markers, we took advantage of a monoclonal antibody Ligab previously generated in our lab using whole Lig-8 cells [17]. The Ligab antibody reacts with the liver progenitor cells Lig-8 but not mature hepatocytes, suggesting that the Lig-8 cells express certain Ligab antigens specific to liver progenitor cells. Moreover, the expression of the Ligab antigens in the Lig-8 cells decreased when the cells underwent SB-induced cell differentiation [17]. Thus, the Ligab antigens could be potential liver progenitor cell markers. Using proteomics, we identified brain isoform glycogen phosphorylase (GPBB) in a protein complex of the Ligab immunoprecipitates from the Lig-8 cells. Immunoblotting showed that GPBB was expressed in the Lig-8 and WB-F344 cells and the levels of GPBB in these cells decreased upon SB-induced cell differentiation, consistent with GPBB as a liver progenitor cell marker. GP is the first enzyme required for glycogenolysis [19]. Our shRNA-mediated GPBB knockdown followed by functional assays shows that GPBB facilitates liver progenitor cell survival under low glucose conditions and SB-induced cell differentiation.

## MATERIALS AND METHODS

### Cell culture and induction of cell differentiation

Lig-8 cells were derived and cultured as previously described [16, 17]. Cells between 29 and 35 passages were used. WB-F344 cells (courtesy of William B. Coleman, University of North Carolina at Chapel Hill, Chapel Hill, NC, USA) [5, 7, 20] were cultured in Dulbecco’s Modified Eagle Medium (DMEM)/F12 containing 10% fetal bovine serum (FBS), 20 mM HEPES (USB Corporation, Cleveland, OH, USA), and 1× penicillin-streptomycin (Invitrogen Corporation, Carlsbad, CA, USA). Cells between 19 and 27 passages were used. Rat liver myofibroblasts (MFs) established previously [20] and rat hepatoma cell line H4IIE (American Type Culture Collection, Manassas, VA, USA) were cultured in DMEM containing 10% FBS. All cells were cultured at 37°C in a humidified atmosphere containing 5% CO_2_. For inducing bi-potential differentiation, WB-F344 cells were cultured in a medium containing 5 mM SB (Sigma-Aldrich, St. Louis, MO, USA) for 1 to 5 days.

### Immunoprecipitation and electrophoresis

As previously described, the Ligab antibody reacts specifically with the Ligab antigen in a non-denaturing protein extraction buffer [17]. Therefore, we prepared Lig-8 cell protein extracts by dounce-homogenizing the cells in a non-denaturing protein lysis buffer containing 1% v/v Triton X-100, 50 mM Tris (pH 7.4), 300 mM NaCl, 5 mM EDTA, 0.02% w/v sodium azide, 1 mM phenylmethylsulfonyl fluoride, and 1% v/v protease inhibitor cocktail (Sigma-Aldrich, St. Louis, MO, USA). The protein extracts were cleared by centrifugation at 12,000 ×*g* at 4°C for 30 minutes and the supernatants were further subjected to ultracentrifugation (Beckman Optima XL-90 Ultracentrifuge, Global Medical Instrumentation Inc., Ramsey, MN, USA) at 226,000 ×*g* at 4°C for 1 hour to separate the cytosolic fraction (S2) from the precipitated membrane fraction (S3). The S2 fraction was further separated into S2.1 (MW > 30 kDa) and S2.2 (MW < 30 kDa) by using a centricon tube (Millipore, Billerica, MA, USA). The S3 membrane precipitates were re-suspended in a non-denaturing lysis buffer containing 0.01% dodecyl-beta-D-maltoside (DDM; Sigma-Aldrich, St. Louis, MO, USA) at 4°C on a rotator overnight followed by centrifugation at 2,000 rpm at 4°C for 10 minutes. The supernatants were then subjected to dialysis to remove the detergent DDM. The protein concentration of each fraction thus obtained was determined using a protein assay dye reagent (Bio-Rad, Hercules, CA, USA).

Each protein fraction thus obtained was incubated with 5 μg of the Ligab antibody and 30 μL of 50% protein G slurry (Invitrogen, Carlsbad, CA, USA) at 4°C on a rotator overnight. The protein G slurries were washed 3 times in the protein lysis buffer and thereafter subjected to standard SDS-PAGE. The gels were silver-stained using SilverSNAP (Thermo Scientific, Rockford, IL, USA).

### Protein identification by using liquid chromatography combined with tandem mass spectrometry

The bands of interest on the silver-stained gels were excised and diced into approximately 1-mm^3^ pieces. The diced gel pieces were washed in microcentrifuge tubes with 100 mM ammonium bicarbonate, dehydrated with 50% acetonitrile (Sigma-Aldrich, St. Louis, MO, USA), and dried completely in a Speed-Vac. The gels were rehydrated and reduced with 50 μL of 10 mM dithiothreitol in 50 μM ammonium bicarbonate. Cysteine residues were alkylated by treating the gels with 50 μL of 55 μM iodoacetamide (Sigma-Aldrich, St. Louis, MO, USA). After the supernatant was washed and decanted, 50 μL of acetonitrile was added to the gels and the gels were dried in a Speed-Vac. The gels were rehydrated in 50 μM ammonium bicarbonate containing sequencing grade trypsin (Roche Diagnostics Ltd., Lewes, UK) at a concentration of 13 ng/μL and incubated at 37°C for 1 hour, and then at room temperature overnight. The final step was to re-dissolve the gel pieces by using a mixture containing 1 μL of 1% formic acid (Sigma-Aldrich, St. Louis, MO, USA) and 9 μL of 50% acetonitrile. The re-dissolved peptide mixture solution was then injected online to a column for liquid chromatography (LC Packings Ultimate, Dionex, Sunnyvale, CA, USA) and thereafter subjected to mass spectrometry (QSTAR XL, Applied Biosystems, Foster City, CA, USA). Protein identification was performed using the MASCOT database search (Matrix Science, Boston, MA, USA). MASCOT scores greater than or equal to 38 indicate identity or extensive homology (p < 0.05).

### Generating and obtaining GP isozyme-specific polyclonal antibodies

Three isoforms exist in mammals, brain isoform glycogen phosphorylase (GPBB), liver isoform glycogen phosphorylase (GPLL) and muscle isoform glycogen phosphorylase (GPMM) [21–23]. Polyclonal antibodies against GPBB and GPLL were generated by immunizing rabbits with peptides synthesized according to the isoform-specific carboxyl-terminal regions: SDLQIPPPNLPKD for GPBB between 831 and 843 and SLSKESSNGVNANGK for GPLL between 836 and 850. The polyclonal antibodies were then purified using affinity chromatography. The GPMM-antiserum has been previously characterized [24].

### Extraction of cellular proteins and immunoblotting analysis

For immunoblotting, Lig-8 and WB-F344 cellular proteins were extracted in a buffer containing 50 mM Tris (pH 8.0), 0.5 mM EDTA, 150 mM NaCl, 0.5% NP-40, and 1× protease inhibitor cocktail (Sigma-Aldrich, St. Louis, MO, USA). Each protein extract (50 μg) in a sample buffer (50 mM Tris (pH 6.8), 2% SDS, 0.05% bromophenol red, and 10% glycerol) was boiled for 10 min, separated on 10% SDS-PAGE, and transferred to a nitrocellulose membrane (Hybond-C extra, Amersham Biosciences, Piscataway, NJ, USA). The membranes were blocked for 4 hours at room temperature in PBS (phosphate-buffered saline: 137 mM NaCl, 2.7 mM KCl, 10 mM Na_2_HPO_4_, 2 mM KH_2_PO_4_) containing 0.05% Tween 20 and 5% skim milk and then incubated overnight at 4°C with primary antibodies (Table 1) in PBS containing 0.05% Tween-20 and 5% skim milk. The membranes were thereafter washed and incubated with horseradish peroxidase-conjugated secondary antibodies at a dilution of 1:10000 for 1 hour at room temperature and then developed using the Western Blot Luminol Reagent Kit (Santa Cruz, Santa Cruz, CA, USA). Protein band intensities in the immunoblots were analyzed using the Image J software (National Institute of Health, Bethesda, MD, USA). For comparison purposes, protein band intensities were normalized against that of the housekeeping protein β-actin.

**Table 1:** Antibodies used for flow cytometry and immunoblotting.

Antibody	Species	Application	Working dilution	Source
Ligab	Mouse monoclonal	FC	1:100	Produced in-house [17]
Rat GPBB	Rabbit polyclonal	IB	1:1000	Produced in-house
Rat GPLL	Rabbit polyclonal	IB	1:1000	Produced in-house
Rat GPMM	Rabbit polyclonal	IB	1:1000	Bernd Hamprecht [24]
Rat CK19	Mouse monoclonal	IB	1:100	Birgit Lane [33, 34]
GAPDH	Mouse monoclonal	IB	1:2000	Millipore
β-actin	Mouse monoclonal	IB	1:15000	Sigma

FC: flow cytometry; IB: Immunoblotting.

### Construction of lentivirus-expressing GPBB-specific short hairpin RNA

Because of the high degree of mRNA homology (87%) among GPBB, GPMM, and GPLL [21–24], we carefully compared the 3 mRNA sequences and selected 2 sequences (nucleotide 3119–3137 and nucleotide 3759–3777), each containing 19 nucleotides that were specific to GPBB. Two pairs of sense and antisense oligonucleotides were synthesized (Table 2). The annealed oligonucleotide pairs were inserted into the BamHI and EcoRI cloning sites of a dual-marker short-hairpin RNA (shRNA) expression vector pGreenPur (System Biosciences, Mountain View, CA, USA). These recombinant vectors were cotransfected with a packaging plasmid, psPAX2, and an envelope plasmid, pMD2G, into HEK293 cells by using JetPEI transfection reagent (Polyplus-transfection Inc., New York, NY, USA) to produce recombinant lentiviruses. The transfection units of the lentiviruses were determined using flow cytometry based on the reporter copGFP. Ten microliters of concentrated viruses were used to infect 10^5^ WB-F344 cells per well of 6-well culture plates. The positively infected cells were selected by treating them with puromycin at 2 μg/mL for 3 days.

**Table 2:** Oligonucleotide sequences of the GPBB shRNAs.

Name of shRNA	Oligonucleotide sequence
shRNA3119	Sense: 5’-GATCC**CCTTTACCCTTGGCTGAAT**cttcctgtcaga**ATTCAGCCAAGGGTAAAGG**TTTTTG-3’
	Antisense: 5’-AATTCAAAAA**CCTTTACCCTTGGCTGAAT**tctgacaggaag**ATTCAGCCAAGGGTAAAGG**G-3’
shRNA3759	Sense: 5’-GATCC**GAGGTCCCTTGAAGCCATA**cttcctgtcaga**TATGGCTTCAAGGGACCTC**TTTTTG-3’
	Antisense: 5’-AATTCAAAAA**GAGGTCCCTTGAAGCCATA**tctgacaggaag**TATGGCTTCAAGGGACCTC**G-3’

Sense strand of the shRNA consists sequentially of an overhanging BamHI, a 19-nucleotide sense target, a loop sequence (lower case), a 19-nucleotide antisense target corresponding to the sense target, and a terminator sequence, TTTTTG. Antisense strand of the shRNA consists of an overhanging EcoRI, an antisense terminator sequence (CAAAAA), a19-nucleotide sense target, a loop sequence (lower case), and the 19-nucleotide antisense target. Bold capital: targeted sense and antisense nucleotide sequences.

### Examination of the Ligab antigen in liver progenitor cells WB-F344 and Lig-8 by flow cytometry

Lig-8 cells have been shown specifically to react with Ligab by flow cytometry and confocal microscope [17]. Here we examined whether WB-F344 cells could also react with Ligab antibody using H4IIE and MF cells as controls. WB-F344, Lig-8, H4IIE, and MF cells were detached from plates, washed 3 times with PBS, and incubated with 5 μg/mL of either Ligab antibody or control mouse IgG on ice for 45 minutes in PBS containing 2 mM ethylenediaminetetraacetic acid (EDTA) and 0.5% bovine serum albumin (BSA). The cells were then washed 3 times with PBS and incubated in PBS containing a 1:100 dilution of fluorescein isothiocyanate-conjugated donkey anti-mouse secondary antibody (Jackson Immunoresearch Laboratories, Baltimore, MD, USA) for 30 minutes in the dark at 4°C. The cells were washed 3 times with PBS in the dark, with each wash lasting 5 minutes. These cells were re-suspended in PBS and analyzed using flow cytometry (BDFACS Calibur, BD Biosciences, San Jose, CA, USA). For examining the Ligab-immunoreactivities of shRNA-mediated GPBB knockdown WB-F344 and Lig-8 cells, Cy5-conjugated secondary antibody was used.

### Apoptotic cell analysis

Apoptotic cell analysis involving propidium iodide (PI) staining was performed using flow cytometry. Lig-8 and WB-F344 cells were cultured in high glucose (4.5 g/L), low glucose (1.0 g/L), or glucose-and-pyruvate-deprived medium at 37°C for 24 hours. For each condition, cells in suspension and cells attached to the dish were harvested, mixed and fixed in 70% ethanol at -20°C for 1 hour. Thereafter, the cells were washed with PBS and stained with PBS containing 20 μg/mL PI, 0.1% Triton X-100, and 0.2 μg/mL DNase-free RNase A in the dark at 4°C for 1 hour. These cells were washed with PBS three times and then analyzed using flow cytometry.

### Statistical analysis

Data were expressed as the mean ± standard error of means from triplicate measurements. Statistical significance were based on Student’s *t* test in Microsoft Excel, set at *P* < 0.05 (*) or *P* < 0.01 (**).

## RESULTS

### Lig-8 and WB-F344 liver progenitor cells express Ligab antigen

**Fig 1:**
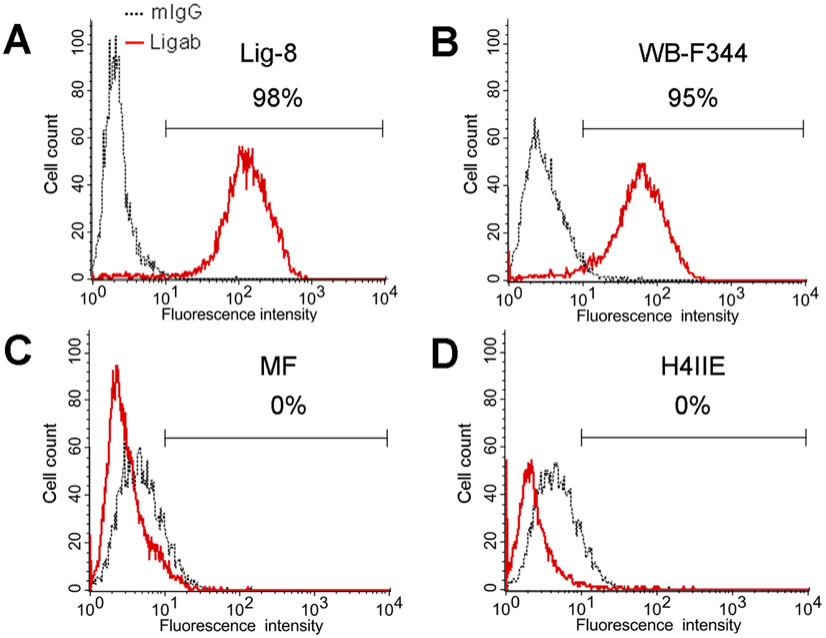
Expression of Ligab antigen in liver progenitor cells. Two liver progenitor cells, Lig-8 (A) and WB-F344 (B) and two liver non-progenitor cells, MF (C) and H4IIE (D) were examined for Ligab antigen expression with flow cytometry. The cells were incubated with the Ligab antibody (red line) or control mouse IgG (grey dotted line) followed by FITC-conjugated secondary antibody staining before flow cytometry analysis. Numbers indicated percent of cells with fluorescence intensity greater than 10^1^.

Before using the monoclonal Ligab antibody to identify potential liver progenitor cell markers, we examined whether this antibody could differentiate liver progenitor cells (Lig-8 and WB-F344) from non-progenitor cells (MF and H4IIE) using flow cytometry. When stained with the Ligab antibody (Fig. 1A, red line), 98.8% of the liver progenitor cells Lig-8 showed staining intensity greater than 10 whereas no apparent staining was found in the Lig-8 cells stained with a non-specific IgG (grey dotted line). Similarly, 95.3% of the liver progenitor cells WB-F344 were stained by the Ligab antibody (Fig. 1B). In contrast, the two non-progenitor cells MF and H4IIE were not stained by the Ligab antibody (Fig. 1C, D). These results suggested that the Ligab antigens expressed in the Lig-8 and WB-F344 cells could be potential markers of liver progenitor cells.

### Proteomics identified glycogen phosphorylase in the Ligab immunoprecipitate

**Fig 2:**
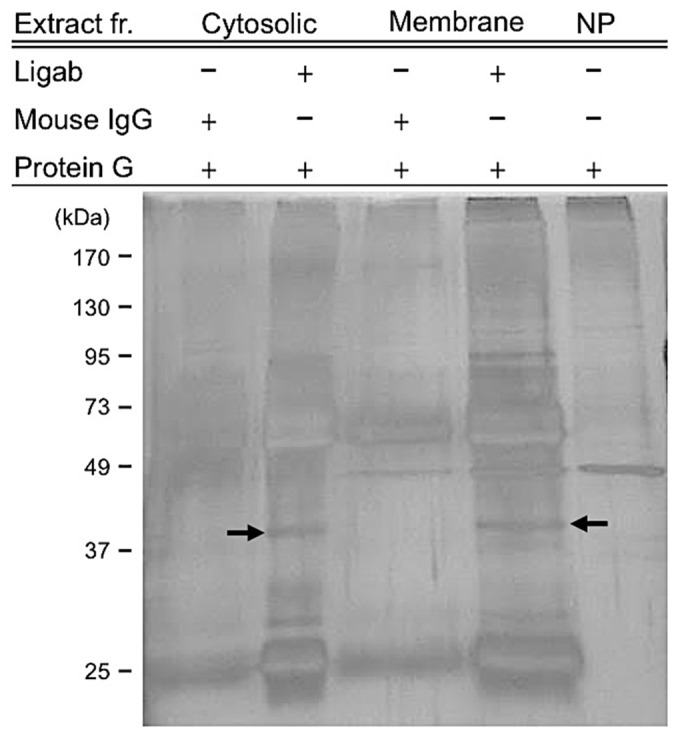
Silver-staining of the Ligab immunoprecipitates of Lig-8 cells. Membrane and cytosolic fractions of the Lig-8 cells were precipitated with the Ligab antibody and subjected to SDS-PAGE separation followed by silver staining. Mouse IgG immunoprecipitates served as a negative control. A unique band at about 38 kDa in the cytosolic and membrane fractions (arrows) were excised and mixed for protein identification by LC-MS/MS. Extract fr.: Extract of protein fraction; NP: no protein.

To identify the Ligab antigens, proteins in the membrane and cytosolic fractions of the Lig-8 cells were immunoprecipitated with the Ligab antibody, separated with SDS-PAGE and silver stained. A unique band at about 38 kDa in the cytosolic and membrane fractions (Fig. 2) were excised and pooled for protein identification by LC-MS/MS. Among 25 peptides identified from this band, a peptide (DFNVGGYIQAVLDR) with a MASCOT score of 110 passing the threshold 38 (p < 0.05) matched glycogen phosphorylase (GP) of *Rattus norvegicus*.

### WB-F344 and Lig-8 cells express brain isoform glycogen phosphorylase

**Fig 3:**
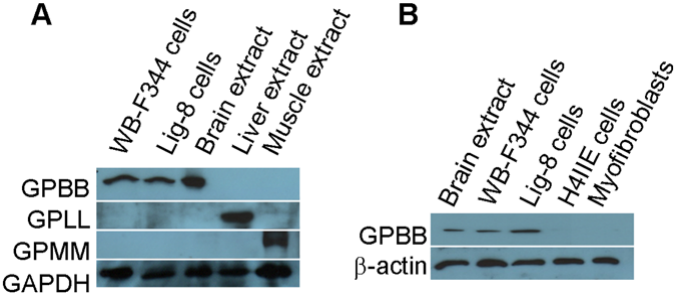
Expression of GP isoforms in liver progenitor cells. Cell lysates from two liver progenitor cells WB-F344 and Lig-8 (A) and two non-progenitor cells MF and H4IIE (B) were analyzed with immunoblotting for the expression of GPBB (brain isoform glycogen phosphorylase), GPLL (liver isoform glycogen phosphorylase) and GPMM (muscle isoform glycogen phosphorylase). Protein extracts from rat brain, liver and muscle served as positive controls for the GPBB, GPLL and GPMM antibodies.

Because there are 3 GP isoforms (GPBB, GPLL and GPMM) in mammals [21–24, 29], we generated isoform-specific antibodies against GPBB and GPLL and acquired an antiserum against GPMM [24] and used them to examine the isoforms expressed in WB-F344 and Lig-8 cells with immunoblotting. As seen in Fig. 3A, only GPBB at about 97 kDa was detected in the WB-F344 and Lig-8 cell lysates whereas GPLL and GPMM were not detectable, indicating that the liver progenitor cells express specifically the brain isoform of glycogen phosphorylase GPBB. In contrast, the non-progenitor cells MF and H4IIE did not express GPBB (Fig. 3B). These results suggested that GPBB is a potential liver progenitor marker. Note the mass difference between GPBB (97 kDa) and the Ligab-immunoprecipitated band (38 kDa) submitted to protein identification.

### GPBB is likely present in a protein complex of the Ligab immunoprecipitate

**Fig 4:**
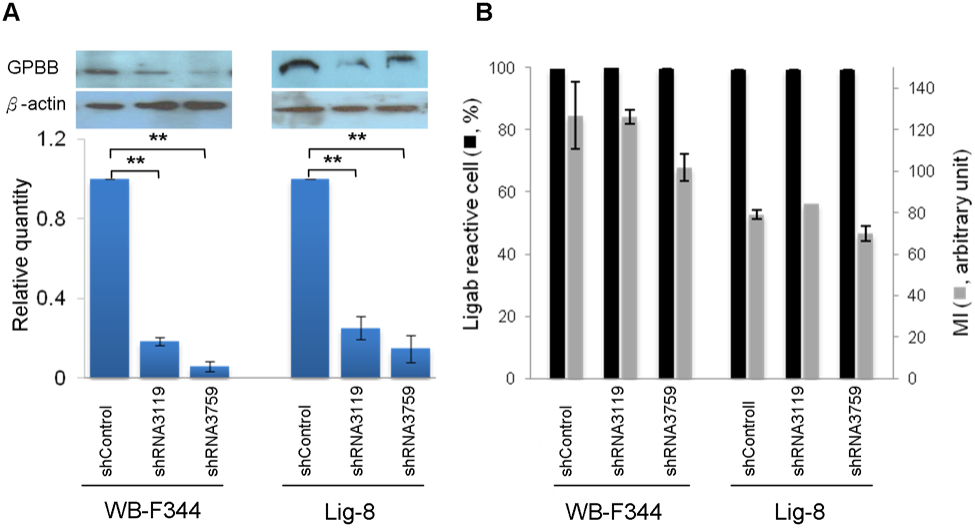
Ligab immunoreactivities in WB-F344 and Lig-8 cells with or without shRNA-mediated GPBB knockdown. (A) Control (shControl) or lentiviruses expressing shRNA (shRNA3119 or shRNA3759) targeting GPBB was used to knockdown GPBB in WB-F344 and Lig-8 cells. After puromycin selection, control and stable GPBB knockdown cells were analyzed for GPBB expression with immunoblotting. The top panel shows the representative immunoblotting results. The bottom panel summarizes the results from 3 experiments. Y-axis: Relative quantity, intensities of protein band over that of shControl. (B) Percentages of Ligab reactive cells and mean fluorescence intensity (MI) by Ligab staining in Lig-8 and WB-F344 cells. Values are mean ± SEM.

**Fig 5:**
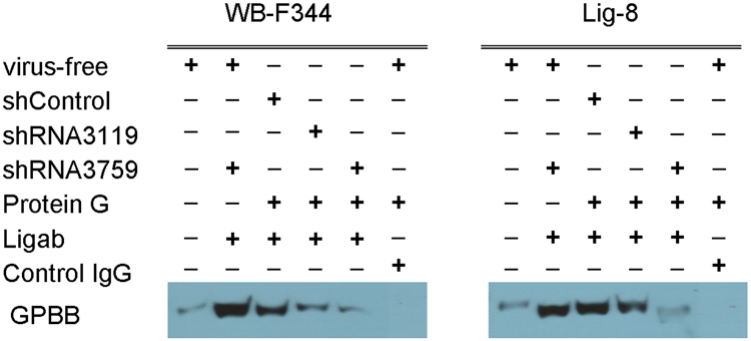
Detection of GPBB in the Ligab immunoprecipitates. Ligab immunoprecipitates were prepared from indicated cell extracts, separated with SDS-PAGE and immunoblotted with the GPBB antibody.

To examine whether GPBB was a specific Ligab antigen, we knocked down GPBB in WB-F344 and Lig-8 cells using shRNA-based method followed by immunoblotting with the GPBB antibody. As seen in Fig. 4A, two shRNA sequences (shRNA3119 and shRNA3759) successfully reduced GPBB protein levels to about 17% and 7% in WB-F344 cells. Similarly, the two shRNA sequences reduced GPBB protein levels to about 36% and 13% in Lig-8 cells. However, GPBB knockdown did not affect reactivity of the Ligab antibody to the GPBB knockdown cells. As shown in Fig. 4B, nearly 100% of the Lig-8 and WB-F344 cells with or without GPBB knockdown still reacted to the Ligab antibody. There was no difference in the mean fluorescence intensity either regardless GPBB knockdown. Thus, GPBB was unlikely a direct Ligab antigen. The reason that GPBB was identified could perhaps be due to its presence in a protein complex immunoprecipitated by the Ligab antibody. In line with this, the Ligab immunoprecipitates from GPBB knockdown cells still showed presence of GPBB albeit at lower amounts (Fig. 5). However, GPBB was not detectable in the control IgG immunoprecipitates, consistent with its presence in the protein complex immunoprecipitated by the Ligab antibody.

### GPBB expression decreased upon sodium butyrate induced cell differentiation

**Fig 6:**
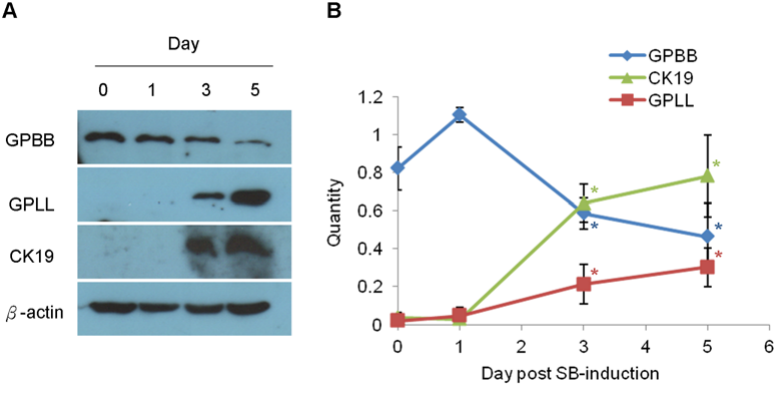
Time course expression of GPBB, GPLL and CK19 in WB-F344 cells during sodium butyrate (SB)-induced differentiation. (A) shows representative immunoblotting results and (B) shows a summary of 3 independent immunoblotting results. The cells were plated onto dishes on day 0 and SB was added into culture conditions on day 1. Y-axis: quantity, intensity of interest protein band over that of β-actin. Values are mean ± SEM. * *P* < 0.05, Student’s test against values of the day 1.

To verify GPBB as a potential liver stem cell marker, sodium butyrate was used to induce WB-F344 cell differentiation followed by examining levels of GPBB protein along with mature cell makers CK19 and GPLL with immunoblotting. Upon sodium butyrate addition to the WB-F344 cells, the mature cell markers CK19 and GPLL were detected on day 3 and continuously increased on day 5 (Fig. 6), indicative of cell differentiation. During the cell differentiation process, GPBB protein levels decreased in a time-dependent manner to about 71% on day 3 and 56% on day 5 compared to those on day 1.

### GPBB knockdown delayed sodium butyrate induced cell differentiation

**Fig 7:**
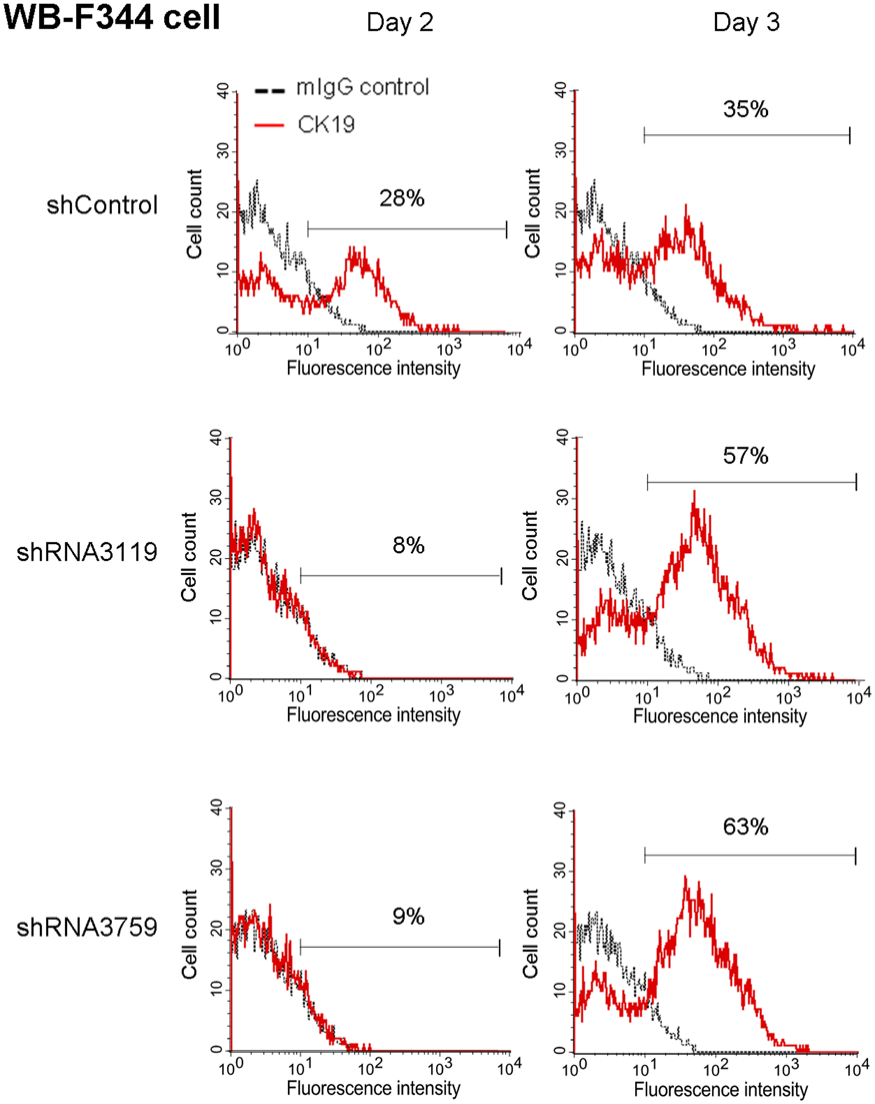
Expression of CK19 in GPBB knockdown WB-F344 cells with sodium butyrate (SB)-induced differentiation. The cells were cultured in the presence of 5 mM sodium butyrate for 2 or 3 days before analyzed for mature cell marker CK19 expression with indirect immunofluorescence staining followed by flow cytometry. Numbers indicate percent of the cells with fluorescence intensity greater than 10^1^. Red line, cells stained with the CK19 antibody; grey dotted line, cells stained with the control mouse IgG.

To investigate potential roles of GPBB in cell differentiation, GPBB was knocked down in WB-F344 cells followed by sodium butyrate induction of differentiation. Fig. 7 shows the flow cytometry analysis results. In the control knockdown cells, sodium butyrate increased the percentage of mature cell marker CK19 positive cells from barely detectable to about 28% on day 2. In contrast, the percentage of CK19 positive cells was only about 8–9% in the GPBB knockdown cells on day 2. After the delay in expressing the CK19 marker on day 2, the percentage of CK19 positive cells in the GPBB knockdown cells reached about 57–63% on day 3 to a level similar to that of the control knockdown cells. These observations suggested that GPBB knockdown delayed sodium butyrate induced WB-F344 cell differentiation.

### GPBB knockdown rendered WB-F344 and Lig-8 cells vulnerable under glucose-deprived conditions

**Fig 8:**
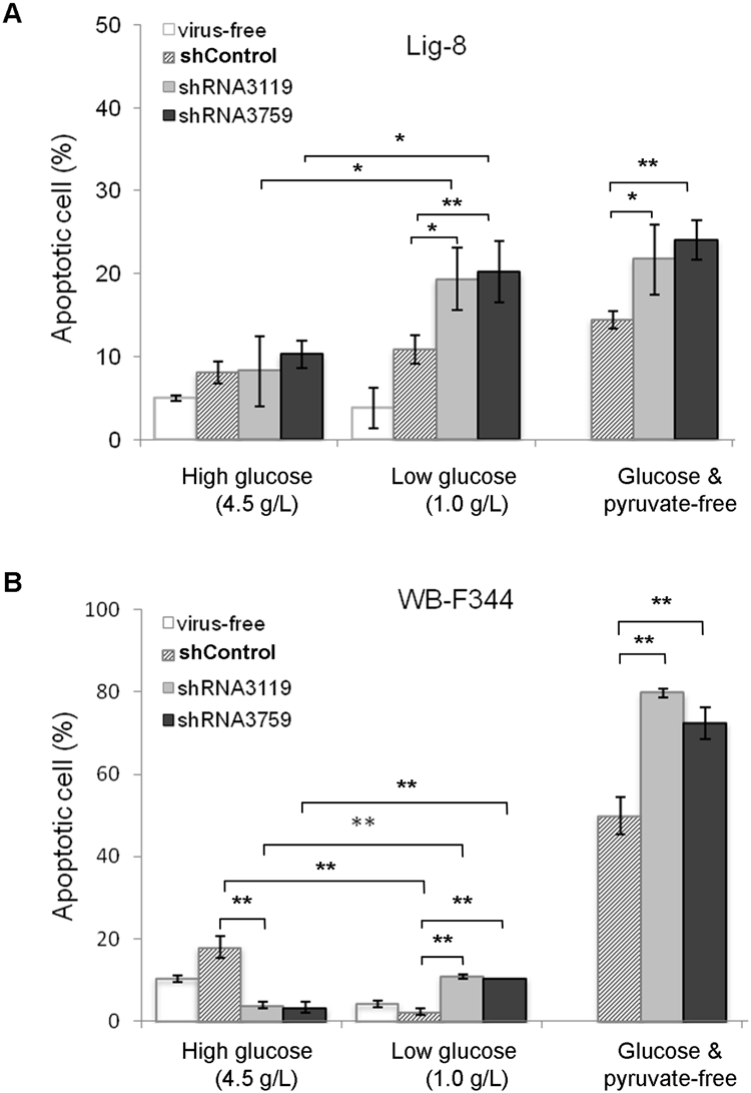
Survival of GPBB knockdown WB-F344 and Lig-8 cells under low glucose conditions. Control or lentivirus expressing shRNA3119 or shRNA3759 targeting GPBB was used to infect Lig-8 (A) and WB-F344 (B) cells. After puromycin selection, stably knockdown cells were cultured in mediums with various levels of glucose: high (4.5 g/L), low (1.0 g/L) or zero glucose/pyruvate for 24 hours. All cells in the supernatant and those attached to the plate were harvested and mixed. The nuclei were stained with propidium iodide before flow cytometry analysis. Cells with nucleus staining intensity less than those of the G_0_/G_1_ phase cells were considered apoptotic. Values are mean ± SEM. * *P* < 0.05 and ** *P* < 0.01, Student’s test against values of the control knockdown (shControl).

Given GP a key enzyme in glycogenolysis, we examined whether GPBB plays a role in liver progenitor cell survival in low glucose medium. Control and GPBB knockdown cells were cultured in medium containing various levels of glucose concentrations followed by flow cytometry analysis for apoptosis. As shown in Fig. 8A, the percentages of the cells undergoing apoptosis were similar (~ 10%) for the control and GPBB knockdown Lig-8 cells in the medium containing 4.5 g/L glucose. When the glucose levels were reduced to 1.0 g/L, the percentages of apoptotic cells significantly increased (19.31% ± 3.77% by shRNA3119 and 20.17% ± 3.70% by shRNA3759 *vs*. 10.81% ± 1.73% by shControl). The percentages of apoptotic cells increased even higher when glucose and pyruvate were both deprived from the medium (21.72% ± 4.24% by shRNA3119 and 24.05% ± 2.41% by shRNA3759 *vs*. 14.41% ± 1.03% by shControl). These results suggest that the liver progenitor cells depend on GPBB for survival under low glucose conditions. Similar results were observed in GPBB knockdown WB-F344 cells under low glucose conditions. The percentages of apoptotic cells were about 4% in medium containing 4.5 g/L glucose (Fig. 8B). In medium containing 1.0 g/L glucose, the apoptotic percentage increased to 10.94% ± 0.52% by shRNA3119 and 10.60% ± 0.06% by shRNA3759 compared to 2.36% ± 0.77% by shControl. When glucose and pyruvate were deprived, more than 70% of the GPBB knockdown WB-F344 cells underwent apoptosis (79.78% ± 1.07% by shRNA3119 and 72.45% ± 3.86% by shRNA3759 *vs*. 49.93% ± 4.47% by shControl). Note that the apoptotic percentage of the control cells was reduced, from 18.03% in high glucose medium (4.5 g/L) to 2.36% in low glucose medium (1.0 g/L).

## DISCUSSION

Despite of many existing liver progenitor cells, the advances in liver regenerative medicine was hampered in part due to the lack of proper cell markers. Taking advantage of a liver progenitor cell specific antibody Ligab generated in our laboratory, we discovered a novel liver progenitor cell marker, GPBB, by analyzing a protein complex of the Ligab immunoprecipitates with LC-MS/MS-based proteomics. GPBB is expressed at a high level in the Lig-8 cell as well as the well-known liver progenitor cell WB-F344 (Fig. 1). Upon SB-induced cell differentiation, GPBB expression levels significantly decrease in WB-F344 cell (Fig. 6), consistent with a role of GPBB as a liver progenitor cell marker. GPBB knockdown followed by glucose deprivation shows that GPBB is required for Lig-8 and WB-F344 cells survival under low glucose conditions (Fig. 8). GPBB knockdown followed by SB-induced cell differentiation shows that GPBB knockdown delays SB-induced cell differentiation (Fig. 7). Our data indicate that GPBB is a liver progenitor cell marker that helps liver progenitor cell survival under low glucose conditions and promotes differentiation.

Although GPBB was identified in the Ligab immunoprecipitates, GPBB is unlikely a direct Ligab antigen for a number of reasons. 1) The Ligab antibody most likely recognizes an unidentified protein in the plasma membrane of the cells because of its ability to detect Lig-8 and WB-F344 whole cells without the use of detergent to permeabilize the plasma membrane (Fig. 1) [17]. GPBB is a cytosolic protein and hence is unlikely a direct Ligab antigen. 2) GPBB knockdown in the Lig-8 and WB-F344 cells did not affect Ligab recognition of the GPBB knockdown cells (Fig. 4). 3) The apparent mass difference between the 38 kDa band submitted to mass spectrometry (Fig. 2) and the 97 kDa GP band detected by our antibody (Fig. 3) suggests an alternative GPBB splice variant. However, our RT-PCR results found no evidence of smaller GPBB splice variants with a protein size close to 38 kDa (data not shown). Thus, the major protein in the 38 kDa band is unlikely GPBB with 97 kDa. How could we identify a 97 kDa protein in a 38 kDa gel piece? Per our experiences in mass spectrometry, it is not uncommon that proteins are identified in gel pieces that do not correspond to the molecular weights of the proteins [25, 26]. This is particularly the case when mild detergents were used in immunoprecipitation experiments. Since GPBB is present in the Ligab immunoprecipitates but not the control IgG immunoprecipitates (Fig. 2), it appears that the Ligab might bind to an as yet unidentified membrane protein of the Lig-8 cells and GPBB somehow co-precipitated along with the Ligab bound protein complex.

The identification of GPBB in the liver progenitor cells was interesting in that GPBB is a fetal-type GP expressed at a high level in early embryonic stages i.e. undifferentiated stages where progenitor cells are in. As the embryos develop, GPBB was gradually replaced by mature forms of GPBB, GPLL and GPMM in the brain, liver and muscles, respectively [21–23, 27–29]. We observed a similar switch in the GP isoforms in the liver progenitor cells undergoing SB-induced cell differentiation. GPBB was expressed at a high level in the WB-F344 cells before SB-induced differentiation (Fig. 6). Upon addition of SB, GPBB started to decrease in the differentiated WB-F344 cells while the mature cell marker GPLL and CK19 began to express (Fig. 6). Consistent with this switch from GPPB to GPLL expression in the differentiated WB-F344 cells, it was interesting to note that GPBB is the predominant GP in poorly differentiated and rapidly growing hepatoma [21] whereas GPLL is the predominant GP in the adult liver cells [30].

GPBB does not seem to maintain stemness of the liver progenitor cells as GPBB knockdown did not induce cell differentiation into either hepatocyte or cholangiocyte lineage (data not shown). Since GP catalyzes the rate-limiting step in glycogenolysis in animals by releasing glucose-1-phosphate from the terminal alpha-1,4-glycosidic bond [28], we suspected roles of GPBB in glycogen metabolism in liver progenitor cells. Our results show that GPBB helps liver progenitor cell survival under low glucose conditions (Fig. 8). GPBB may also facilitate liver progenitor cell differentiation because GPBB knockdown delayed SB-induced cell differentiation (Fig. 7). These results suggest that extracellular glucose concentrations affect liver progenitor cell survival and differentiation. Similar observations were made in human tenocytes where extracellular glucose concentrations were reported to determine cell fate following oxidative stress [31]. GPBB expression, however, is not necessarily a favorable factor for the liver progenitor cells under high glucose conditions which cause a high percentage of apoptotic cells (Fig. 8B). Similarly, high glucose concentrations were reported to cause apoptosis of mesenchymal stem cells [32].

We conclude for the first time that GPBB is a novel liver progenitor cell marker. Expression of GPBB in the liver progenitor cells appears to play dual roles in facilitating liver progenitor cell survival under low glucose conditions and cell differentiation.
